# Orbital Shift‐Induced Boundary Obstructed Topological Materials with a Large Energy Gap

**DOI:** 10.1002/advs.202202564

**Published:** 2022-07-29

**Authors:** Ning Mao, Runhan Li, Ying Dai, Baibiao Huang, Binghai Yan, Chengwang Niu

**Affiliations:** ^1^ School of Physics State Key Laboratory of Crystal Materials Shandong University Jinan 250100 China; ^2^ Department of Condensed Matter Physics Weizmann Institute of Science Rehovot Israel

**Keywords:** boundary obstruction, corner states, higher‐order topology, hinge states, second‐order topology, surface states, third‐order topology

## Abstract

Boundary obstructed topological phases caused by Wannier orbital shift between ordinary atomic sites are proposed, which, however, cannot be indicated by symmetry eigenvalues at high symmetry momenta (symmetry indicators) in bulk. On the open boundary, Wannier charge centers can shift to different atoms from those in bulk, leading to in‐gap surface states, higher‐order hinge states or corner states. To demonstrate such orbital shift‐induced boundary obstructed topological insulators, eight material candidates are predicted, all of which are overlooked in the present topological databases. Metallic surface states, hinge states, or corner states cover the large bulk energy gap (e.g., more than 1 eV in TlGaTe_2_) at related boundary, which are ready for experimental detection. Additionally, these materials are also fragile topological insulators with hourglass‐like surface states.

## Graphical abstract

The authors put forward t he boundary obstructed topological phases caused by Wannier orbital shift between ordinary atomic sites. Moreover, a general principle to trigger the higher‐order states is proposed. Armed with this method, five material candidates are predicted for second‐order and three for third‐order topological insulators, all of which are overlooked in the present topological databases.

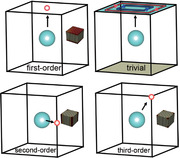

## 1 Introduction

While boundary conditions play rich implications for both fundamental physics and potential applications, the boundary obstruction is currently introduced for the topological classification of quantum matters, where even if two Hamiltonians are adiabatically equivalent under periodic boundary conditions, they cannot be connected for certain surface terminations under open boundary conditions.^[^ ^1^, ^2^, ^3^, ^4^ ^]^ Different from the conventional topological insulators (TIs),^[^ ^5^, ^6^, ^7^, ^8^, ^9^ ^]^ the boundary obstructed topological insulators (BOTIs) are not captured by bulk energy gap closing in periodic boundary conditions, revealing a hidden topology of overlooked trivial insulators.^[^ ^10^, ^11^, ^12^, ^13^, ^14^, ^15^, ^16^, ^17^ ^]^ Indeed, topology of conventional trivial insulators currently matures into a significant burgeoning field, describing an extension and fine‐graining of the topological paradigm.^[^ ^18^, ^19^, ^20^ ^]^ For instance, obstructed atomic insulators (OAIs) and orbital‐selected OAIs (OOAIs) form as results of the spatial obstruction and representation obstruction, respectively.^[^ ^21^ ^]^ And remarkably, they have emerged as promising candidates for many interesting properties including low work function, strong hydrogen affinity, and electrocatalysis.^[^ ^22^, ^23^ ^]^ Moreover, the boundary‐obstructed topological high‐temperature superconductivity is proposed in iron pnictides, opening a new arena for highly stable Majorana modes.^[^ ^1^, ^2^ ^]^

Characterized with generalized bulk‐boundary correspondence, on the other hand, higher‐order TIs have expanded the topological classification and recently drawn significant attentions.^[^ ^24^, ^25^, ^26^, ^27^, ^28^, ^29^, ^30^, ^31^, ^32^, ^33^, ^34^ ^]^ For which, the *n*th‐order TIs in *d* dimensions host‐protected features, such as hinge or corner states, at (*d* − *n*)‐dimensional boundaries. Currently, both the second‐ and third‐order TIs have been experimentally confirmed in a variety of metamaterials.^[^ ^35^, ^36^, ^37^, ^38^, ^39^, ^40^ ^]^ However, for electronic materials, realizations of higher‐order TIs are limited to second‐order ones, and the third‐order TI is still missing. Therefore, a general method toward predicting higher‐order TIs with realistic material candidates is highly desirable. Interestingly, systems having boundary obstructions are characterized either by hinge or corner states, arising as a result of quantized electric quadrupole or octupole moments.^[^ ^4^, ^41^, ^42^ ^]^ Such electric multipole moments origin from the Wannier orbitals shift between an occupied Wyckoff position and empty Wyckoff position for the BOTIs. Therefore, a natural question arises as to whether the Wannier orbitals shift between occupied Wyckoff positions, previously regarded trivial phase, can result in the emergence of hidden topology and even with either hinge or corner states.

**Figure 1:**
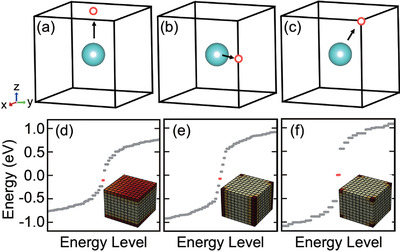
Sketch of the boundary states versus the boundary polarization. The unit cell contains one cyan atom at the center of cubic lattice, and Wannier orbital of atom shift from cyan atoms to red circle along the direction of black arrow. When the Wannier orbital shift into a) plane, b) hinge, and c) corner, the d) surface states, e) hinge states, and f) corner states emerge, respectively.

In the present work, we put forward the realization of orbital shift‐induced BOTIs with fragile topology, namely boundary obstructed fragile TIs (BOFTIs). As a consequence of boundary obstruction, metallic surface states and higher‐order hinge or corner states can emerge regardless of spin–orbit coupling (SOC) or bulk band inversion. Under periodic boundary condition, BOFTIs exhibit trivial symmetry indicators and trivial real‐space invariants.^[^ ^10^, ^43^ ^]^ Under certain open boundary condition, at least one of the Wannier orbitals will deviate from the original Wyckoff position and shift into another occupied Wyckoff position, giving rise to induced surface states. Based on the topological quantum chemistry (TQC) theory which is actually beyond symmetry indicators,^[^ ^10^ ^]^ we provide a general principle to generate the higher‐order states, which depend on the boundary polarization direction, by comparing the occupied outer‐shell Wannier orbitals in compounds with free atomic orbitals (see **Figure** **1**). Furthermore, we identify eight experimentally feasible material candidates of BOFTIs by using the first‐principles calculations in space group *I*4/*mcm*. Besides the metallic surface states, five of them possess symmetry‐protected hinge states while the other three exhibit nontrivial corner states, rendering them the second‐ and third‐order TIs, respectively.

## 2 Results

**Figure 2:**
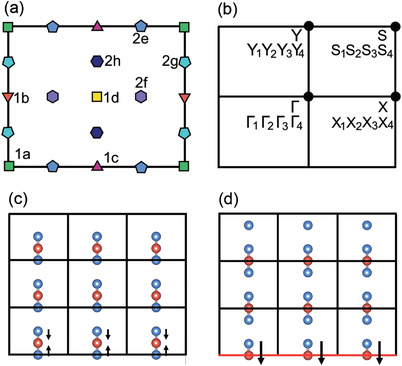
a) Wyckoff positions of the space group *pmm*2. b) Brillouin zone with the decomposition of Bloch bands into irreducible representations. Real‐space configurations terminated for c) blue and d) red atoms.

We demonstrate the orbital shift‐induced BOTI with a cubic space group *Pmm*2 (No.25) or equivalently the layer group *pmm*2 (No.23), which is generated by two symmetry operations, $C_{2z}\left(x\to -x,\;y\to -y\right)$ and $M_{y}\left(y\to -y\right)$. There are four inequivalent *C*_2*z*_ invariant maximal Wyckoff positions, 1a, 1b, 1c, and 1d as illustrated in **Figure** **2**a. Besides, there are four $M_{x/y}$ invariant non‐maximal Wyckoff positions, 2e, 2f, 2g, and 2h. For an atomic insulator, both the Bloch and Wannier states are trapped in the Wyckoff positions.^[^ ^44^ ^]^ The atomic orbital μ on a Wyckoff position j is denoted as |Rjμ⟩ with **R** being the position of unit cell, and the Wannier state of |Rjμ⟩ is constructed by the Fourier transformation, $|W_{R,\alpha }\rangle =\sum_{R^{\prime }j\mu }S_{j\mu ,\alpha }\left(R-R^{\prime }\right)\left|R^{\prime }j\mu \right\rangle$. In the following, we will show that boundary obstruction can also give rise to surface and higher‐order states, forming into a novel quantum state of BOFTI. Under periodic boundary condition, BOFTI have the same symmetry characters as atomic insulators who have localized Wannier charge centers (WCCs) at the corresponding atom. Therefore, BOFTI cannot be identified by the symmetry indicators or real‐space invariants.^[^ ^12^, ^45^, ^46^ ^]^ However, on the open boundary condition, the WCCs of BOFTI will shift into another orbital that does not belong to the original orbital, giving rise to a nonzero boundary polarization. When the boundary polarization is vertical to the plane, metallic surface states may show up in this plane. While, if the boundary polarization is vertical to the hinge or corner of the structure, symmetry‐protected hinge states or corner states manifest as a result of electric quadrupole or octupole moment (see Figure 1 and Figures S1‐S5, Supporting Information).

Here, we consider three atoms, which are located at two Wyckoff positions as 1d (1/2, 1/2, 1/2) and 2h (1/2, ±y, 1/2). Without considering the effects of SOC, $T^{2}$ = 1, the single‐valued irreducible representations (irreps) for 1d are A_1_, A_2_, B_1_, B_2_ and for 2h are $A^{\prime }$, $A^{\prime \prime }$ (see Tables 1‐2, Supporting Information). The $A^{\prime }$ and $A^{\prime \prime }$ are mutually conjugated under the symmetry $C_{2z}$, meaning that, if we put an atomic orbital that transforms as irreps $A^{\prime }$ at (1/2, y, 1/2), denoted as |0*hA*′〉_*L*_, there must be another atomic orbital located at (1/2, ‐y, 1/2) transforms as irreps $A^{\prime \prime }$, denoted as |0*hA*″〉_*R*_. Given the fact that group *m* is a subgroup of *mm*2, the subduction and induction relations can be deduced as follows:

(1) $$\begin{array}{ccc} A_{1}↓m & = & A^{\prime }\\ A_{2}↓m & = & A^{\prime \prime }\\ B_{1}↓m & = & A^{\prime \prime }\\ B_{2}↓m & = & A^{\prime }\\ A^{\prime }↑mm2 & = & A_{1}+B_{2}\\ A^{\prime \prime }↑mm2 & = & A_{2}+B_{1} \end{array}$$

Such relations explicitly show us that all four atomic orbitals at 1d, $|W_{0dA_{1}}\rangle$, $|W_{0dA_{2}}\rangle$, $|W_{0dB_{1}}\rangle$, and $|W_{0dB_{2}}\rangle$, can adiabatically move to 2h and form two atomic orbitals ${|W_{0hA^{\prime }}\rangle }_{L}$ and ${|W_{0hA^{\prime \prime }}\rangle }_{R}$. For which, any one of the four Wannier functions cannot be reduced to preserve the equivalence of 2h and 1d. Therefore, a real‐space invariant is defined as^[^ ^18^, ^19^, ^45^ ^]^

(2) $$\begin{array}{c} \delta_{1}=m\left(A_{1}\right)+m\left(B_{2}\right)-m\left(A_{2}\right)-m\left(B_{1}\right) \end{array}$$

At first, we put four isolated atomic orbitals 2|0*hA*′〉, 2|0*hA*″〉 in the Wyckoff position 2h, and they will spread to form four separated energy bands with elementary band representations (EBRs) 2*A*′@2*h* and 2*A*″@2*h* in the momentum space. These four bands correspond to the natural atomic limit with Brillouin zone (BZ) decomposition as illustrated in Figure 2b, and give rise to a combination of EBRs as 2*A*′@2*h* + 2*A*″@2*h* = Γ_1_ + Γ_2_ + Γ_3_ + Γ_4_ + *X*_1_ + *X*_2_ + *X*_3_ + *X*_4_ + *Y*_1_ + *Y*_2_ + *Y*_3_ + *Y*_4_ + *S*_1_ + *S*_2_ + *S*_3_ + *S*_4_. When atoms are brought together to form a crystal and the Wannier states move to 1d as assumed, the ${|W_{0dA_{1}}\rangle }_{L},{|W_{0dA_{2}}\rangle }_{L},{|W_{0dB_{1}}\rangle }_{L}$, and ${|W_{0dB_{2}}\rangle }_{R}$ are formed with a combination of EBRs *A*_1_@1*d* + *A*_2_@1*d* + *B*_1_@1*d* + *B*_2_@1*d* = Γ_1_ + Γ_2_ + Γ_3_ + Γ_4_ + *X*_1_ + *X*_2_ + *X*_3_ + *X*_4_ + *Y*_1_ + *Y*_2_ + *Y*_3_ + *Y*_4_ + *S*_1_ + *S*_2_ + *S*_3_ + *S*_4_. Clearly, these Wannier states share the same symmetry characters with the isolated ones, and thus leads to the same trivial symmetry indicators and trivial real‐space invariant δ_1_ = 0. Therefore, one can see that BOTIs can be adiabatically equivalent to an atomic insulator in periodic boundary conditions. However, remarkably, metallic boundary states can still be obtained even in the absence of SOC as a consequence of open boundary condition, giving rise to the exotic nontrivial topology.

To show this explicitly, two distinct boundary terminations are considered. While the vertical and horizontal edges lie at *x* = 0 and *y* = 0 that intersect at Wyckoff position 1a, as illustrated in Figure 2c, the Wannier states can deform from 2h to 1d similar to the periodic bulk state as discussed above. Every ${|W_{RhA^{\prime }}\rangle }_{L}$ is connected with a ${|W_{RhA^{\prime \prime }}\rangle }_{R}$ passing through the 1d. Since two positions of 2h are not distinguished, there is no boundary polarization, leading to a trivial termination without any boundary state. On the other hand, without loss of generality, when the edges intersect at 1b with *x* = 0 and *y* = 1/2 shown in Figure 2d, the opposite horizontal edges are distinctly different owing to the fact that the Wyckoff position 1d cannot be shared by two unit cells on opposite edges. That means the red electrons emerge only on one horizontal edge (*y* = 1/2, marked with red line), but not on the opposite one (*y* = 7/2). The absence of ${|W_{y=-1hA^{\prime }}\rangle }_{L}$ obstruct the Wannier state at 2h to adiabatically evolve to the 1d, leading to an unavoidable Wannier state at 1d. Considering the initial atomic orbital, along the direction tangent to the edge *y* = 1/2, the boundary polarization emerges, which gives rise to the exotic edge states serving as a hallmark of BOFTI. Thus, BOFTIs represent a nontrivial phase with localized Wannier functions, while manifest surface states depending on the boundary terminations.

**Figure 3:**
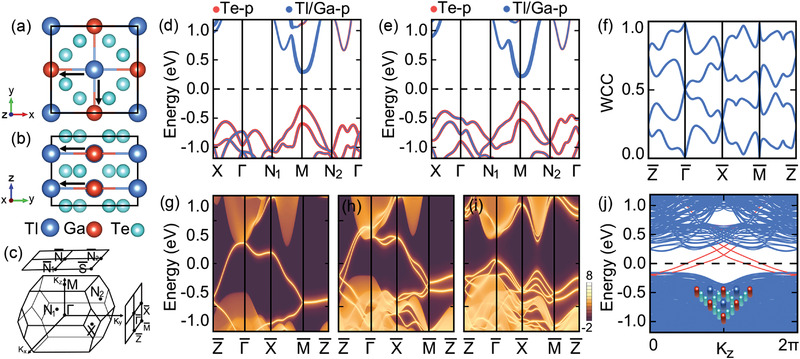
a) Top and b) side views of TlGaTe_2_ in space group *I*4/*mcm*. c) Brillouin zones for the bulk, (001), and (010) surfaces. Orbitally resolved band structures for TlGaTe_2_ d) without and e) with SOC, weighted with the contribution of Te‐*p* and Tl/Ga‐*p* states. The Fermi level is indicated with a black dashed line. f) The [010]‐directed Wilson loop spectra for the top four isolated valence bands. Metallic surface states of semi‐infinite (010) surface g) without and h) with SOC, terminated with the Tl‐Ga‐Tl atoms. i) Gaped surface states of semi‐infinite (010) surface with SOC, terminated with the Te–Te atoms. j) The band structure of TlGaTe_2_ nanorod along k_*z*_ with the hinge modes indicated in red colors. Inset shows the boundary termination of nanorod.

Armed with the above definition, we then establish the material realization of BOFTIs with second‐order topology in space group *I*4/*mcm* (No. 140). **Figure** **3**a,b presents the top and side views of ternary thallium chalcogenide, TlGaTe_2_, which crystallizes in the tetragonal structure with eight atoms in the primitive unit cell. Two Tl atoms occupy the Wyckoff position 4a, two Ga atoms occupy the 4b, and four Te atoms occupy the 8h.^[^ ^47^, ^48^ ^]^ Each Ga atom is surrounded by four nearest‐neighboring Tl atoms within the same layer. The orbitally resolved band structures of TlGaTe_2_ without and with SOC are illustrated in Figure 3d,e. The band gaps are 581 and 433 meV, respectively, with the band contribution remaining almost the same around the Fermi level before and after inclusion of SOC, which enlarge to 1.17 and 1.03 eV as further checked by the hybrid functional calculations.^[^ ^49^ ^]^ One important aspect to highlight here is that there is no SOC‐induced band inversion, that is, band gap closing and reopening process, in TlGaTe_2_, revealing that there is no topological phase transition and thus exhibits the same topological character for TlGaTe_2_ without and with SOC.

According to TQC theory and WCC calculations (see Figure S6, Supporting Information), the occupied bands of TlGaTe_2_ are a combination of EBRs, which can be written as *A*_1_@4*a* + *A*_1_@4*b* + *B*_2_@4*b* + *E*@4*b* + *A*_1_@8*h* + *B*_1_@8*h*. Therefore, the occupied outer‐shell atomic orbitals for Tl, Ga, and Te are *s* ^2^, *s* ^2^ *p* ^6^, and *s* ^2^ *p* ^2^, respectively. Compared to the *s* ^2^ *p* ^1^, *s* ^2^ *p* ^1^, and *s* ^2^ *p* ^4^ for Tl, Ga, and Te elements, the electron transfer occurs from Tl‐*p* orbital to Ga‐*p* orbital. Remarkably, such an electron‐transfer process contributes a boundary polarization with both x and y components, implying that TlGaTe_2_ is potentially a BOFTI with metallic surface states on the (100) and (010) surfaces, and even with the second‐order nontrivial topology.

To confirm the band topology, we implement the direct calculations of WCCs along *k*_*x*_‐, *k*_*y*_‐, and *k*_*z*_‐directions by the Wilson loop method.^[^ ^50^ ^]^ Localized groups of Wilson bands are obtained (see Figure S6, Supporting Information), indicating trivial symmetry indicators of both the $Z_{2}$ and $Z_{8}$ invariants. The trivial Wilson bands are consistent with the analysis of EBR, all serving as a signal of atomic insulator for TlGaTe_2_. Besides, the BR of top four isolated valence bands is a combination of EBRs with positive integer, different from the negative coefficient reported in refs. [51, 52]. However, as shown in Figure 3f, the nontrivial winding of Wilson loop along a bent path demonstrates the fragile topological nature of theses four isolated bands.^[^ ^53^, ^54^ ^]^

Remarkably, the metallic surface states of TlGaTe_2_ always emerge regardless of the SOC, as shown in Figure 3g,h, although the specific band dispersions are different with respect to T and glide mirror symmetry $G_{x}=\left\{M_{x}|00\frac{1}{2}\right\}$. Along $G_{x}$ invariant lines $\bar{\Gamma }\bar{Z}$ and $\bar{M}\bar{X}$, one has $G_{x}^{2}=e^{-ik_{z}}$ in the absence of SOC, and thus the eigenvalues of $G_{x}$ will be $g_{\pm x}=\pm e^{-ik_{z}/2}$, namely ±1 at $\bar{\Gamma }$, $\bar{X}$ and ±*i* at $\bar{Z}$, $\bar{M}$. Meanwhile, the T imposes further constraints, guaranteeing the Kramers degeneracy of $\bar{Z}$ and $\bar{M}$. Moreover, the surface bands along $\bar{M}\bar{Z}$ are always doubly degenerate due to the protection of $TG_{x}$. Consistent with our model analysis, the surface nodal line appears only on one surface but vanishes on the opposite one as a result of the boundary obstruction (see Figure S7, Supporting Information). After taking SOC into consideration, the glide mirrors satisfy $G_{x}^{2}=-e^{-ik_{z}}$, results in the eigenvalues of $g_{\pm x}=\pm ie^{-ik_{z}/2}$, that is, ±1 at $\bar{Z}$, $\bar{M}$ and ±*i* at $\bar{\Gamma }$, $\bar{X}$. In this case, the partner switching of Kramers pairs between $\bar{\Gamma }$ and $\bar{Z}$ ($\bar{M}$ and $\bar{X}$) enforces a band crossing, constituting the so‐called hourglass surface states as presented in Figure 3h. Interestingly, when glide mirror symmetry remains under perturbations, the exotic hourglass‐like boundary states are quite robust and survive even they are no longer connecting the conduction and valance bands (see Figure S9, Supporting Information). Moreover, due to the phase factor of glide mirror symmetry, the periodicity becomes 4π, rather than 2π for a mirror symmetry. The formed surface states are called as Möbius states (see Figure S16, Supporting Information). Besides, the Dirac crossings near the $\bar{\Gamma }\bar{Z}$ are projected into two intertwined rings (see Figure S8, Supporting Information). It is noted that the spin is locked to the carrier momentum, rendering the spin–momentum locking effect. While, two non‐degenerate rings with the left‐handed and right‐handed helicities emerge simultaneously in one surface, giving rise to a trivial Berry phase when electrons encircle the two rings in the surface Brillouin zone. Since $G_{x/y}$ only protect Dirac crossing of (010/100) surface, (110), (1$\bar{1}$0), and ($\bar{1}10$) surface will be gapped. Therefore, hinge states emerge at the intersection of the (110) and ($\bar{1}$10) surface, via bending the (010) surface along [010] direction.^[^ ^25^, ^54^, ^55^ ^]^ Due to the existence of $G_{x}$, gapped (110) and ($\bar{1}$10) surface will share opposite Dirac mass terms, leading to nonsymmorphic symmetry‐protected hinge states as exhibited in Figure 3j.

**Figure 4:**
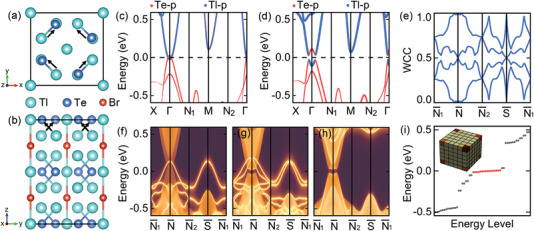
a) Top and b) side views of Tl_5_Te_2_Br in space group *I*4/*mcm*. Orbitally resolved band structures c) without and d) with SOC, weighted with the contribution of Te‐*p* and Tl‐*p* states. The Fermi level is indicated with a black dashed line. e) The [001]‐directed Wilson loop spectra for the top four isolated valence bands. (001)‐surface band structure of Tl_5_Te_2_Br calculated using surface Green's functions for top surface f) without or g) with SOC, terminated with Tl–Te–Te–Tl–Te–Te–Tl atoms. h) Gaped surface states of (001) surface for bottom surface with SOC, terminated with Tl–Tl–Tl–Tl atoms. i) Energy levels of a finite lattice composed of 6 × 6 × 6 conventional unit cells for Tl_5_Te_2_Br, terminated with Tl–Tl, Tl–Tl, and Tl–Br–Tl–Br–Tl atoms. Inset shows the probability of corner states, where we sum all contributions of probability in one unit cell to represent with a cubic.

We then employ the Tl_5_Te_2_Br as an example to demonstrate the feasibility of BOFTI with third‐order topology. For which, 10 Tl atoms occupy the 4c and 16l, 4 Te atoms occupy the 8h, and 2 Br atoms occupy the 4a^[^ ^47^, ^48^ ^]^ as shown in **Figure** **4**a,b. The orbitally resolved band structures of Tl_5_Te_2_Br without and with SOC are displayed in Figure 4c,d, respectively. In the absence of SOC, Tl‐p and Te‐p orbitals overlap and form a closed nodal line around the Γ point, revealing a band inversion with the Tl‐*p* being lower than Te‐*p*. The SOC gaps out the nodal line and drives the Tl_5_Te_2_Br to be an insulator with an energy gap of 56 meV. For this situation, a band inversion with opposite parities, that is, different irreps, is usually obtained and considered as a heuristic scenario of nontrivial bulk topology. However, the irreps of valence band maximum (VBM) at Γ are the same with that of the conduction band minimum (CBM), resulting in a band inversion without irreps inversion and thus a trivial symmetry indicator. The valence bands can be decomposed into a linear combination of EBR

(3) $$\begin{array}{c} \begin{array}{cc} A_{1}@4a+ & A_{2}@4a+E@4a+A_{g}@4c+2A_{1}@8h+\\ & B_{1}@8h+B_{2}@8h+A^{\prime }@16l \end{array} \end{array}$$

suggesting the occupied outer‐shell atomic orbitals of *s* ^2^, *s* ^2^ *p* ^6^, and *s* ^2^ *p* ^6^ for Tl, Te, and Br atoms, respectively. Therefore, the electrons of Tl‐*p* are transferred to both the Te‐*p* and Br‐*p* owing to the outer‐shell atomic orbitals for respective elements are *s* ^2^ *p* ^1^, *s* ^2^ *p* ^5^, and *s* ^2^ *p* ^4^. Interestingly, the electron transfer from 16l to 8h contributes an body‐diagonal polarization, which may give rise to the BOFTI with third‐order topology as discussed in our model analysis.

The fragile nature of Tl_5_Te_2_Br is identified by the Wilson loop spectra of isolated top four valence bands along a bent path as shown in Figure 4e. Furthermore, Figure 4f–h shows us metallic surface states, exhibiting exotic wallpaper fermions such as hourglass fermion, fourfold‐degenerate Dirac fermion, as a consequence of two perpendicular glide mirrors, $G_{x/y}=\left\{M_{x/y}|\frac{1}{2}\frac{1}{2}0\right\}$. Similar to the case of TlGaTe_2_, the Fermi surface of Tl_5_Te_2_Br still presents us two rings with the left‐handed and right‐handed helicities in one surface (see Figure S8, Supporting Information). Then, we explore the corner states, which is the hallmark of a 3D third‐order TI. To this end, we focus on a finite lattice composed of 6 × 6 × 6 conventional unit cell, and adopt the open boundary conditions for all the [100], [010], and [001] directions. As shown in Figure 4i, 16 nearly degenerate in‐gap states arise around the Fermi level. Notably, wave functions of the in‐gap states are localized almost at eight corners and vanishing in other region.

## 3 Discussion

Inside the 454 materials of the TQC website which belong to space group *I*4/*mcm* (No. 140), 8 of them are predicted to BOFTIs with the implementation of the first‐principles calculations.^[^ ^57^ ^]^ Among them, five materials such as *X*GaTe_2_ (*X* = Tl, In, and Na), BaPbO_3_, and InTe manifest metallic surface states on the (100) and (010) surface, exhibiting symmetry‐protected hourglass fermion. In addition, the BOFTIs can co‐exist with the normal TI, which achieved in the InGaTe_2_. While the surface states of normal TIs show up in both the top and bottom surfaces at the same time, the surface states of BOFTIs can only emerge in one surface (see Figure S10, Supporting Information). Interestingly, along the intersection lines of (110) and ($\bar{1}10$) surface, two pairs of hinge states arise as a hallmark of SOTI. Moreover, three materials such as Tl_5_Te_2_Br and Tl_4_Se_3_ *Y* (*Y* = Pb and Sn) show surface states only on the (001) surface with exotic hourglass fermion, and fourfold‐degenerate Dirac fermion. The 16 nearly degenerate corner states only arise from the intersection of (100), (010), and (001) surfaces.

## 4 Conclusion

In conclusion, we demonstrate the co‐existence of BOFTIs and higher‐order topological phase with both the metallic surface states and hinge states or corner states. Remarkably, the emergence of surface states is a consequence of boundary obstruction, rather than the SOC, realizing the nontrivial characters in previously overlooked trivial insulators. Using TlGaTe_2_ and Tl_5_Te_2_Br as two examples, we show that tangent and body‐diagonal boundary polarizations result in the birth of second‐ and third‐order TIs, respectively, as identified by the energy spectrum calculations of nanorod and finite lattice. Especially, TlGaTe_2_ exhibits a bulk energy gap more than 1 eV, which provides advantages for experiments to detect obstructed boundary states on the surface and edge. Our study lay the groundwork of finding exotic topological phases through boundary obstruction, and promote it as a new platform for exploring the intriguing physics of exotic surface states and fragile topological phases.

## Conflict of Interest

The authors declare no conflict of interest.

## Supporting information

Supporting Information

## Data Availability

The data that support the findings of this study are available from the corresponding author upon reasonable request.
