# Supplementary material for: Orbital Shift‐Induced Boundary Obstructed Topological Materials with a Large Energy Gap
Source: Adv Sci (Weinh). 2022 Jul 29;9(27):2202564. doi: 10.1002/advs.202202564 (PMC9507389; doi:10.1002/advs.202202564)
Supplement: Supplementary file 1 — Supporting Information [file ADVS-9-2202564-s001.pdf]

ADVANCED SCIENCE
Open Access

Supporting Information

for *Adv. Sci.*, DOI 10.1002/advs.202202564

Orbital Shift-Induced Boundary Obstructed Topological Materials with a Large Energy Gap

Ning Mao, Runhan Li, Ying Dai, Baibiao Huang, Binghai Yan and Chengwang Niu**

Orbital shift-induced boundary obstructed topological materials with a giant energy gap

Ning Mao,[1] Runhan Li,[1] Ying Dai,[1,*] Baibiao Huang,[1] Binghai Yan,[2] and Chengwang Niu[1,†]

[1]*School of Physics, State Key Laboratory of Crystal Materials, Shandong University, Jinan 250100, China*
[2]*Department of Condensed Matter Physics, Weizmann Institute of Science, Rehovot, Israel*

Contents

I. Calculation methods 2

II. Model results about the relation between boundary polarizations and higher-order topological phases 2

III. Character table of point group m (C_s) and $mm2$ (C_{2v}) 3

IV. Band structures, Wilson loop spectra and energy level of finite lattice for the corresponding normal insulator, TI, SOTI, and TOTI. 4

V. The spin texture, surface states and Wilson loop spectra of $TlGaTe_2$ and Tl_5Te_2Br 9

VI. The geometry structures, bulk bands, Wilson bands, surface bands and nanorod bands of the SOTI material candidates 13

VII. The geometry structures, bulk bands, Wilson bands, surface bands, energy level of finite lattice and probability of corner states of TOTI material candidates 17

VIII. Schematic demonstration for the spin transport and Möbius states. 19

IX. Surface fermion under perturbations. 20

X. Detailed crystallographic information and previous growth reports of the eight candidates 21

References 21

I. Calculation methods

All the structures file of $I4/mcm$ (No.140) are downloaded from www.topologicalquantumchemistry.com/. The first-principles calculations are carried out in the framework of generalized gradient approximation (GGA) with Perdew–Burke–Ernzerhof (PBE) [1] functionals using the Vienna Ab initio simulation package (VASP) [2] and the full-potential linearized augmented-plane-wave method using the FLEUR code. The cutoff energy for the plane-wave basis set was 500 eV. A Γ-centred 8×8×6 k-mesh was found to be suitable to sample the Brillouin zone for both the structural relaxations and self-consistent total energy calculations, whereas the maximum force on every atom falls below 0.01 eV/Åand the difference in energy of successive electronic steps are less than 10-6 eV. The maximally localized Wannier functions (MLWFs) are constructed using the Wannier90 code [3] in conjunction with the FLEUR package [4, 5] and the surface states calculations are using WannierTools [6]. The symmetry eigenvalues and related irreducible representations are calculated using vasp2trace code, which adapts the notation on the Bilbao Crystallographic server.

II. Model results about the relation between boundary polarizations and higher-order topological phases

To find a general method towards higher-order topological insulators (HOTIs), we shall reformulate its concept in a Wannier description. In general, Wannier description of an boundary obstructed fragile topological insulator (BOFTI) would admit a localized behavior and respect all symmetries, whose Wannier states are localized to the boundary atoms representing an obstruction to localize into original positions. This is intuitively seen by recognizing that behaviour as an electron transferred process. Besides, such a process is always followed by an electric polarization. Which means, an electric polarizations will come into being whose dipole moment is equal to a multiplications of electric charge and distance between electron and original atoms. Limited by crystal symmetry, the bulk electric polarizations often are quantized to some specific values, while the different kinds of boundary may exhibit different kinds of edge polarizations. Such an edge polarization can only take in four different forms: (i) electrons are transferred to the bulk else except boundary, resulting in a vanishing edge polarization; (ii) nonzero edge polarizations tangent to the edge; (iii) the direction of edge polarizations is along body-diagonal; and (iv) edge polarizations has components of three directions. Aboved four forms correspond to the formation of four topological states like bulk states, surface states, hinge states, and corner states, respectively, where we mainly focus on the higher-order topological phases as forms (ii), (iii) and (iv).

To be explicit, let us consider an eight-band BHZ model. We give a detailed description of the relationship between edge polarizations and four topological phase, with a Hamiltonian expressed as

$$\begin{aligned} H(\mathbf{k}) &= \lambda_y \sin(k_y)\,\Gamma'^1 + [\gamma_y + \lambda_y \cos(k_y)]\,\Gamma'^2 \\ &+ \lambda_x \sin(k_x)\,\Gamma'^3 + [\gamma_x + \lambda_x \cos(k_x)]\,\Gamma'^4 \\ &+ \lambda_z \sin(k_z)\,\Gamma'^5 + [\gamma_z + \lambda_z \cos(k_z)]\,\Gamma'^6, \end{aligned} \tag{1}$$

with $\Gamma'_0 = \sigma_3 \otimes \tau_3 \otimes \mu_0$, $\Gamma'_1 = \sigma_3 \otimes \tau_2 \otimes \mu_1$, $\Gamma'_2 = \sigma_3 \otimes \tau_2 \otimes \mu_2$, $\Gamma'_3 = \sigma_3 \otimes \tau_2 \otimes \mu_3$, $\Gamma'_4 = \sigma_3 \otimes \tau_1 \otimes \mu_0$, $\Gamma'_5 = \sigma_2 \otimes \tau_0 \otimes \mu_0$, $\Gamma'_6 = i\Gamma'_0\Gamma'_1\Gamma'_2\Gamma'_3\Gamma'_4\Gamma'_5$. Here, σ_j, τ_j, and μ_j are three Pauli matrices corresponding to eight cyan atoms in a unit cell as shown in Figure. S1. Depending on the relative strength of λ_i and γ_i, electric polarizations p_i will be incorporated by means of a large λ_i with i being x, y, or, z. Likewise, if λ_i is larger than γ_i, a nontrivial polarizations with a magnitude of 1/2 will be generated along i direction. Therefore, atomic insulator will emerge once we chose $\lambda_i < \gamma_i$ (i = x, y, and, z). Under this circumstance, no any nontrivial nature such as surface states, hinge states, corner states will show up, since electrons are closely bound to the corresponding atoms.

With the Hamitonian of a unit cell above, we set $\lambda_x = \lambda_y = \gamma_z = 0.5$, $\gamma_x = \gamma_y = \lambda_z = 1.0$ and diagonalize the Hamiltonian of a $10 \times 10 \times 10$ finite lattice under an open boundary condition. The energy spectrum in Figure. S3(b) exhibits gapless in-gap states within the bulk gap, which is distinct from the atomic insulators. To identify the surface states, we calculate the wave function probability of in-gap states, which suggests surface states only localized on the (001) surface, as displayed in the Figure. S3(c). In Figures. S3(d)- S3(f), the Wannier charge center (WCC) of z direction has a crossing point with $\nu_z = 1/2$, representing a tangential polarization of $\mathbf{p} = (p_x, p_y, p_z) = (0, 0, 1/2)$.

To transform the surface states to the hinge states of SOTI, we have to introduce a face-diagonal polarization, where we set $\gamma_x = \gamma_y = \lambda_z = 0.5$, $\lambda_x = \lambda_y = \gamma_z = 1.0$. Upon the calculation of WCC of Figures. S4(d)- S4(f), we obtain a value of $\mathbf{p} = (1/2, 1/2, 0)$, representing a face-diagonal polarization. As the electrons are enforced to settle in the up and down surfaces,

we suppose a phase transition towards OAI and SOTI can happen. It's noted that the energy spectrum of a $10 \times 10 \times 10$ finite lattice for the OAI and SOTI are similar to each other except that in-gap states of SOTI are more linear near the Fermi level, as shown in Figure. S4(b). However, the distributions of wave function are totally different. As shown in the inset of Figure. S4(c), the in-gap states are symmetrically localized at the four hinges, which usually serve as a evidence of a SOTI phase.

In addition to the two cases discussed above, we also consider another case that both directions have edge polarizations. To achieve that, we shall set $\gamma_x = \gamma_y = \gamma_z = 0.5$, $\lambda_x = \lambda_y = \lambda_z = 1.0$. The WCC calculations in Figures. S5(d)- S5(f) are also in agreement the body-diagonal polarization. Under such a polarization, electrons have nowhere to go but only to the the corners of the unit cell. To testify the existence of TOTI, it's straightforward to diagonalize the Hamiltonian of a $10 \times 10 \times 10$ finite lattice under an open boundary condition. In Figure. S5(b), the energy spectrum only exhibit limited in-gap states within the bulk gap up to eight, which is obviously distinct from the previous three cases. Remarkably, by further analysing the distributions of wave function, we found that eight in-gap states are localized at eight corners, as shown in the inset of Figure. S5(c).

The model results indicate that, the different nonvanishing edge polarizations can serve as a signal of HOTI. Likewisely, hinge states will emerge if edge polarizations only have two components of directions, while corner states only exist for a body-diagonal polarization. Thus, the question about how to find HOTIs is transformed into the question about how to determine the polarizations. To resolve this problem, we seek help for the TQC theory. According to this theory, given the Wyckoff positions and the orbital symmetry of electron, all symmetry characters of atomic insulators can be gained, consistuting the so-called elementary band representation (EBR). An insulator whose occupied states can be represented as a combination of EBR is called as atomic insulator, which is equivalent to a set of symmetric exponentially decayed Wannier functions. Besides, EBR can also be seen as a molecule orbital in a solid, which means that occupied EBR are equivalent as occupied molecule orbitals. Let us reconsider the model lattice in Figure. S1, where eight A' orbitals located on the Wyckoff position 8r in space group P4/mmm. If the occupied bands transform as $A'@8r$, which indicate that no electrons are transferred to anywhere and polarization are totally zero no matter for bulk or boundary. While, if we decompose the occupied bands to get an EBR such as $A_1@4j$, $A_1@2g$, or $A_{1g}@1a$, we can predict that a nontrivial phase is obtained with a manifestation of surface state, hinge state, or corner state.

III. Character table of point group m (C_s) and $mm2$ (C_{2v})

TABLE I: Traces of the single valued irreps of the point groups m (C_s)

C_s	E	$\mathcal{M}_{010}$
A'	1	1
A''	1	-1

TABLE II: Traces of the single valued irreps of the point groups $mm2$ (C_{2v})

C_{2v}	E	2_{001}	$\mathcal{M}_{100}$	$\mathcal{M}_{010}$
A_1	1	1	1	1
A_2	1	1	-1	-1
B_2	1	-1	1	-1
B_1	1	-1	-1	1

IV. Band structures, Wilson loop spectra and energy level of finite lattice for the corresponding normal insulator, TI, SOTI, and TOTI.

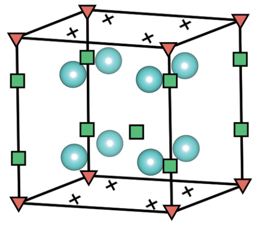

Figure S 1. Sketch of a three-dimensional (3D) cubic lattice, in which eight cyan atoms are located at Wyckoff position 8r of space group P4/mmm (No.123). The black crisscross, green rectangle, and red triangle denote for possible position of electron, which correspond to Wyckoff position 4j, 2g and 1a.

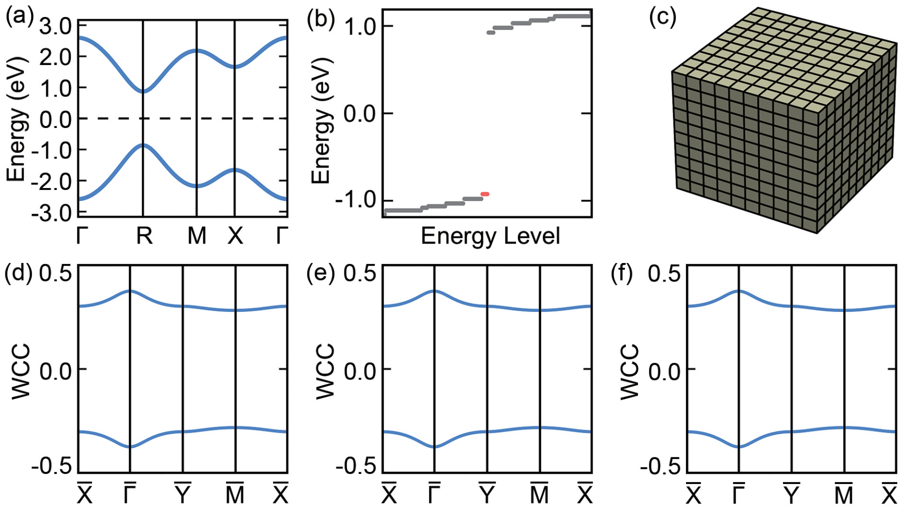

Figure S 2. (a) Band structures under periodic boundary condition along x, y, and z axis. (b) Energy levels of a finite lattice composed of $10 \times 10 \times 10$ conventional unit cells. (c) Probability of red in-gap states. (d)-(f) Wannier charge centers along x, y, and z axis, exhibiting a polarization of (0, 0, 0).

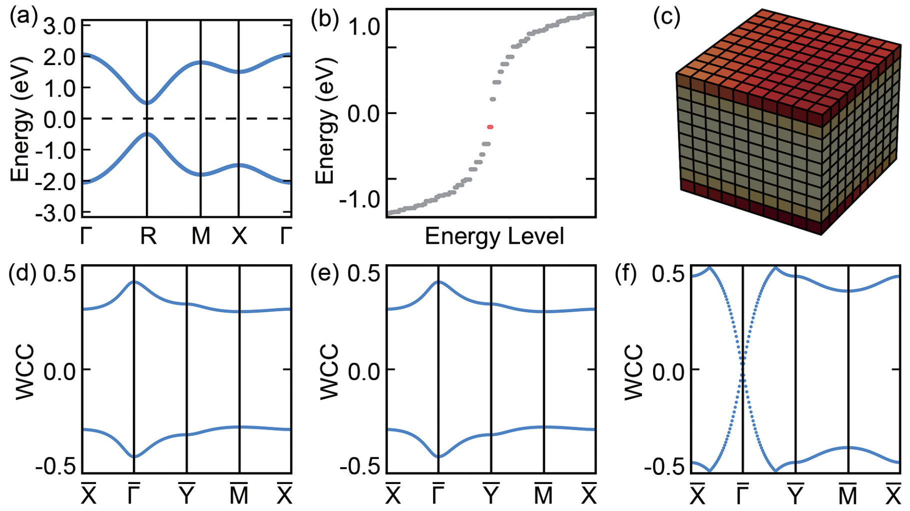

Figure S 3. (a) Band structures under periodic boundary condition along x, y, and z axis. (b) Energy levels of a finite lattice composed of $10 \times 10 \times 10$ conventional unit cells. (c) Probability of red in-gap states. (d)-(f) Wannier charge centers along x, y, and z axis, exhibiting a polarization of (0, 0, 1/2).

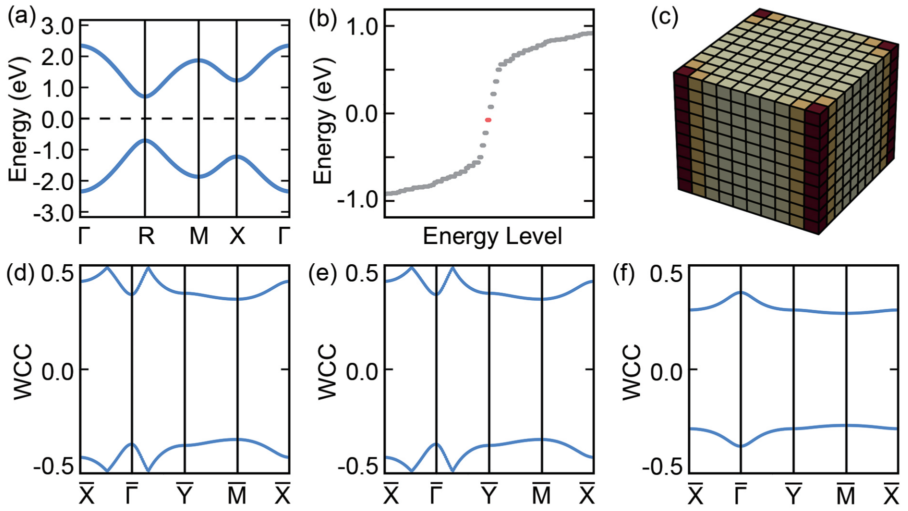

Figure S 4. (a) Band structures under periodic boundary condition along x, y, and z axis. (b) Energy levels of a finite lattice composed of $10 \times 10 \times 10$ conventional unit cells. (c) Probability of red in-gap states. (d)-(f) Wannier charge centers along x, y, and z axis, exhibiting a polarization of (1/2, 1/2, 0).

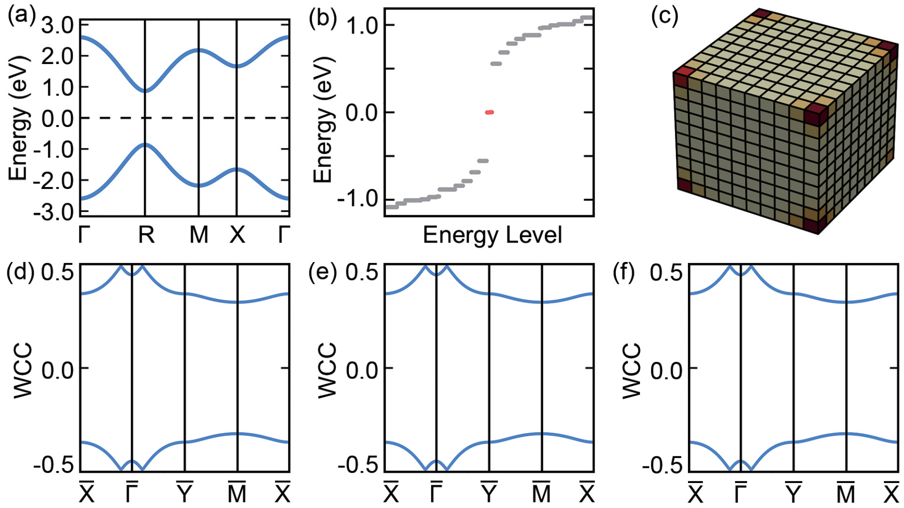

Figure S 5. (a) Band structures under periodic boundary condition along x, y, and z axis. (b) Energy levels of a finite lattice composed of $10 \times 10 \times 10$ conventional unit cells. (c) Probability of red in-gap states. (d)-(f) Wannier charge centers along x, y, and z axis, exhibiting a polarization of (1/2, 1/2, 1/2).

V. The spin texture, surface states and Wilson loop spectra of $TlGaTe_2$ and Tl_5Te_2Br

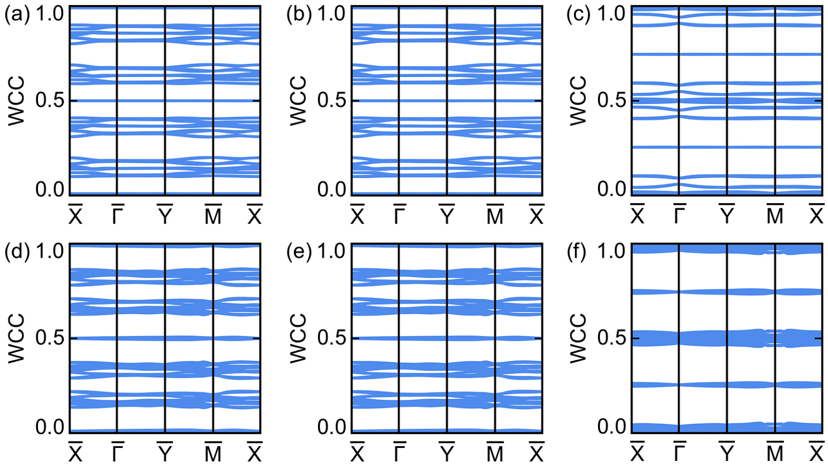

Figure S 6. (a)-(c) Wilson bands of the $TlGaTe_2$ along x, y, and z axis, giving rise to a phase of atomic insulator. (d)-(f) Wilson bands of the Tl_5Te_2Br along x, y, and z axis, giving rise to a phase of atomic insulator.

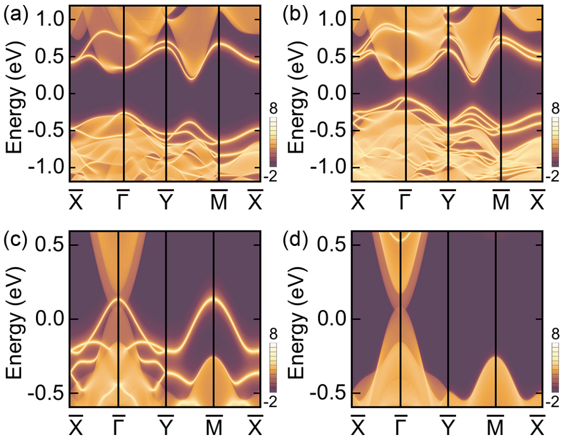

Figure S 7. The down surface of (010)-surface band structure for $TlGaTe_2$ (a) without and (b) with SOC, calculated using surface Green's functions. The Fermi level is set to zero. The (c) up and (d) down surface of (010)-surface band structure for Tl_5Te_2Br without SOC, calculated using surface Green's functions. The Fermi level is set to zero.

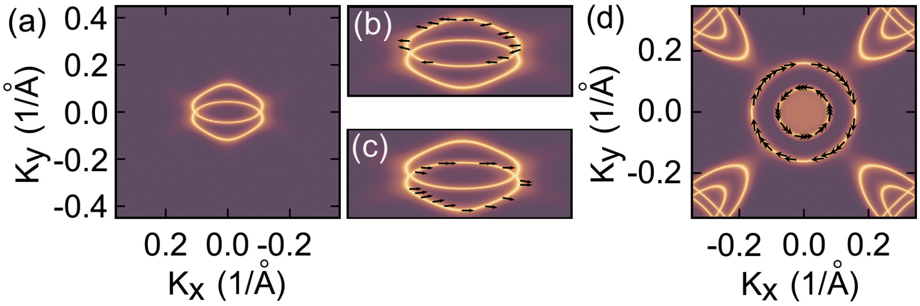

Figure S 8. (a) Fermi surface of the (010)-surface states at -0.02 eV energy contour for $TlGaTe_2$, emerging two intertwined rings in the surface Brillouin zone. (b)-(c) Spin texture for the upper and lower rings. The anticlockwise spin orientation of upper ring implies a right-handed helicity, while the corresponding clockwise spin orientation of lower ring implies a left-handed helicity. (d) Spin texture of the (001)-surface states at 0.06 eV energy contour for Tl_5Te_2Br, where two rings surround the Γ point of the surface Brillouin zone. The outer and inner rings indicate a left-handed and right-handed helicities, respectively.

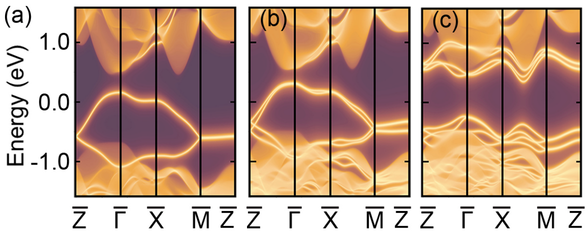

Figure S 9. Metallic surface states (a) without, (b) with SOC, and gapped surface states of semi-infinite (010)-surface, checked by hybrid functional calculations.

VI. The geometry structures, bulk bands, Wilson bands, surface bands and nanorod bands of the SOTI material candidates

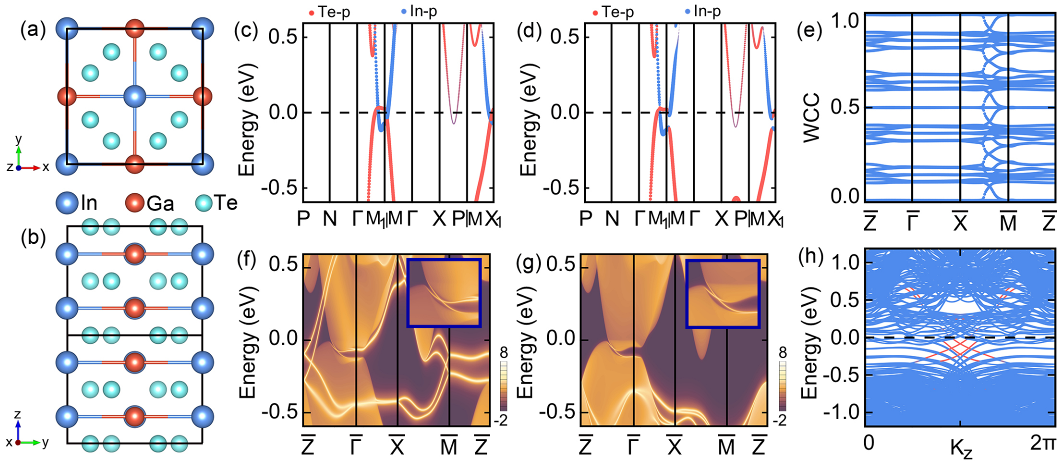

Figure S 10. (a) Top and (b) side views of $InGaTe_2$ conventional unit cell. Bulk band structures of $InGaTe_2$ (c) without and (d) with SOC. The bands are orbitally weighted with the contribution of Te-p and In-p states. The Fermi level is indicated with a black dashed line. (e) The Wilson bands of (010)-surface, where we take all occupied valence bands for the calculations. The (010)-surface band structures of $InGaTe_2$ for (f) top and (g) bottom surfaces, where the insets present the magnified topological insulator's surface states. The Fermi level is set to zero. (h) The Band structure of the nanorod along k_z where the hinge modes are indicated with red colors.

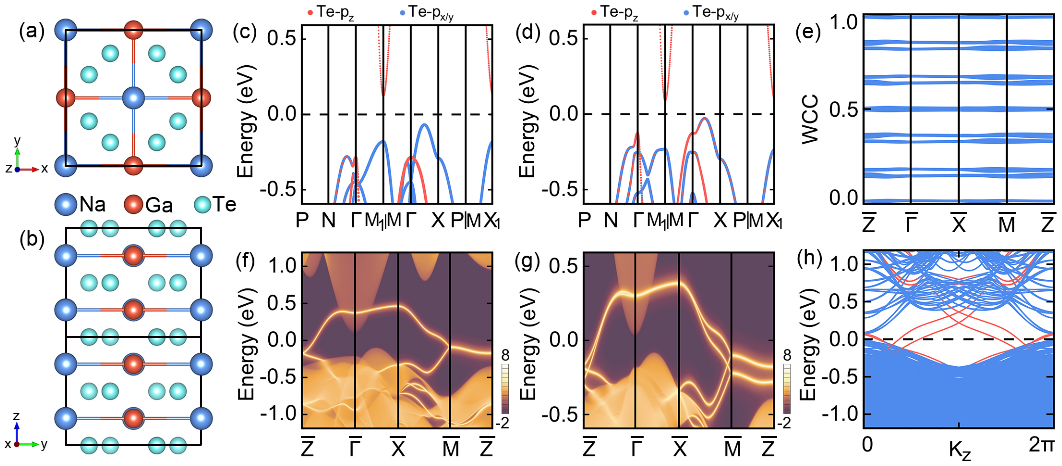

Figure S 11. (a) Top and (b) side view of $NaGaTe_2$ conventional unit cell. Bulk band structures of $NaGaTe_2$ (c) without and (d) with SOC. The bands are orbitally weighted with the contribution of Te-p_z and In-p_x/y states. The Fermi level is indicated with a black dashed line. (e) The Wilson bands of (010)-surface, where we take all occupied valence bands for calculations. The (010)-surface band structure of $NaGaTe_2$ (g) without and (h) with SOC, calculated using surface Green's functions. The Fermi level is set to zero. (h) The band structure of the nanorod along k_z where the hinge modes are indicated with red colors.

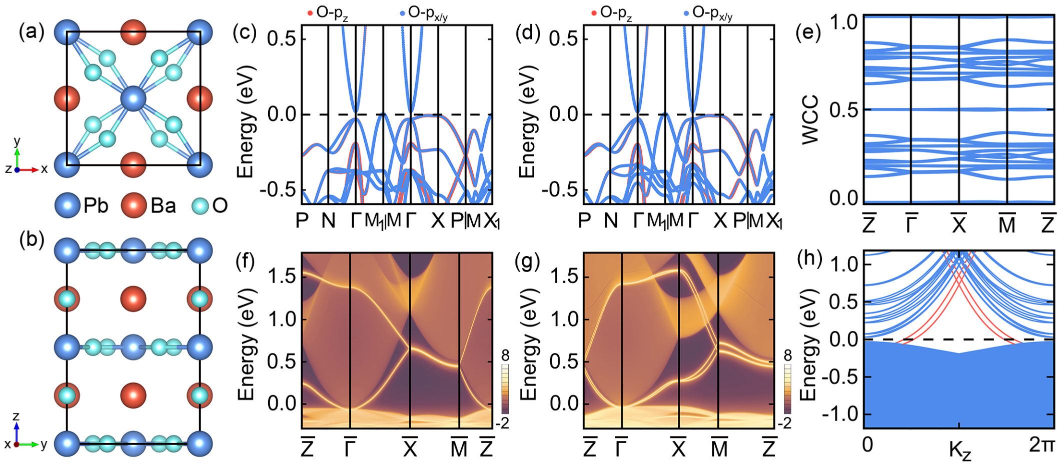

Figure S 12. (a) Top and (b) side view of $BaPbO_3$ conventional unit cell. Bulk band structures of $BaPbO_3$ (c) without and (d) with SOC. The bands are orbitally weighted with the contribution of O-p_z and O-p_x/y states. The Fermi level is indicated with a black dashed line. (e) The Wilson bands of (010)-surface, where we take all occupied valence bands for calculations. The (010)-surface band structure of $BaPbO_3$ (g) without and (h) with SOC, calculated using surface Green's functions. The Fermi level is set to zero. (h) The band structure of the nanorod along k_z where the hinge modes are indicated with red colors.

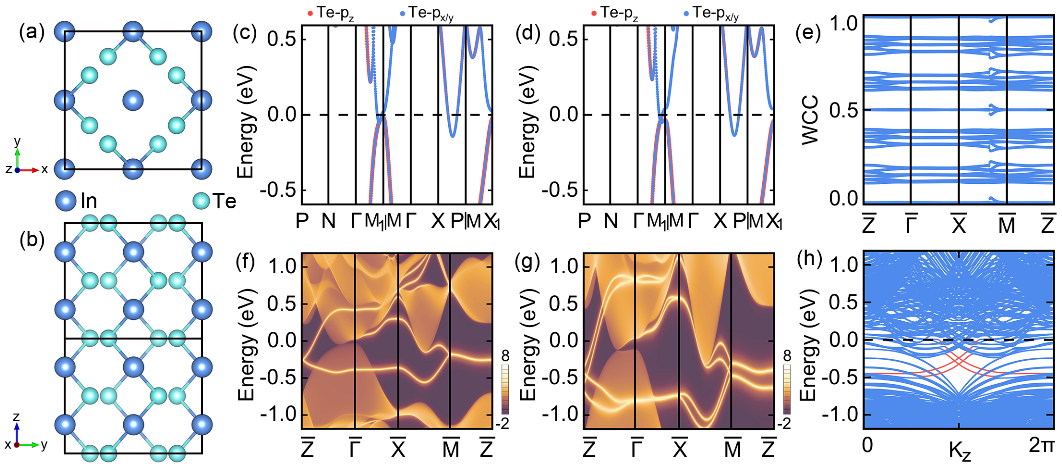

Figure S 13. (a) Top and (b) side view of InTe conventional unit cell. Bulk band structures of InTe (c) without and (d) with SOC. The bands are orbitally weighted with the contribution of Te-p$_z$ and Te-p$_{x}/y$ states. The Fermi level is indicated with a black dashed line. (e) The Wilson bands of (010)-surface, where we take all occupied valence bands for calculations. The (010)-surface band structure of InTe (g) without and (h) with SOC, calculated using surface Green's functions. The Fermi level is set to zero. (h) The band structure of the nanorod along k$_z$ where the hinge modes are indicated with red colors.

VII. The geometry structures, bulk bands, Wilson bands, surface bands, energy level of finite lattice and probability of corner states of TOTI material candidates

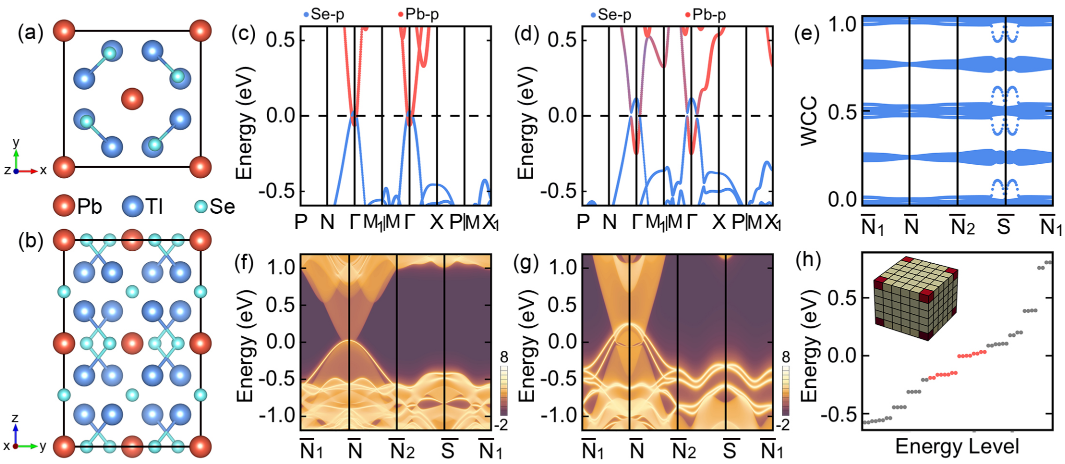

Figure S 14. (a) Top and (b) side view of Tl_4Se_3Pb conventional unit cell. Bulk band structure of Tl_4Se_3Pb (c) without and (d) with SOC, where the bands are orbitally weighted with the contributions of Se-p and Pb-p states. The Fermi level is indicated with a dashed line. (e) The Wilson bands of (001)-surface, where we all occupied valence bands for calculations. (001)-surface band structure of Tl_4Se_3Pb calculated using surface Green's functions (f) without and (g) with SOC. The Fermi level is set to zero. (h) Energy levels of a finite lattice composed of $6 \times 6 \times 6$ conventional unit cells for Tl_4Se_3Pb. There are 16 degenerate corner states as indicated with red color. Insets show the probability of corner states, where we sum all contributions of probability in one unit cell to represente with a cubic.

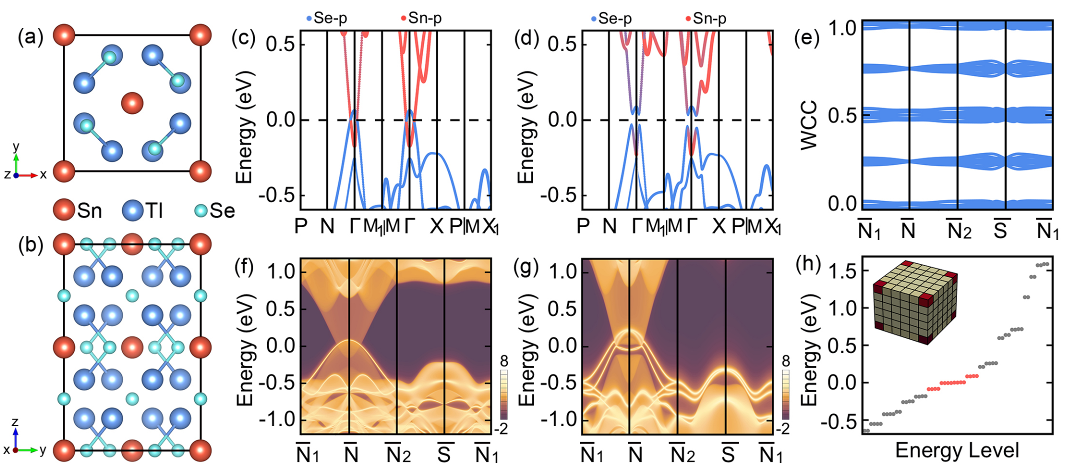

Figure S 15. (a) Top and (b) side view of Tl_4Se_3Sn conventional unit cell. Bulk band structure of Tl_4Se_3Sn (c) without and (d) with SOC, where the bands are orbitally weighted with the contributions of Se-p and Sn-p states. The Fermi level is indicated with a dashed line. (e) The Wilson bands of (001)-surface, where we all occupied valence bands for calculations. (001)-surface band structure of Tl_4Se_3Sn calculated using surface Green's functions (f) without and (g) with SOC. The Fermi level is set to zero. (h) Energy levels of a finite lattice composed of $6 \times 6 \times 6$ conventional unit cells for Tl_4Se_3Sn. There are 16 degenerate corner states as indicated with red color. Insets show the probability of corner states, where we sum all contributions of probability in one unit cell to represente with a cubic.

VIII. Schematic demonstration for the spin transport and Möbius states.

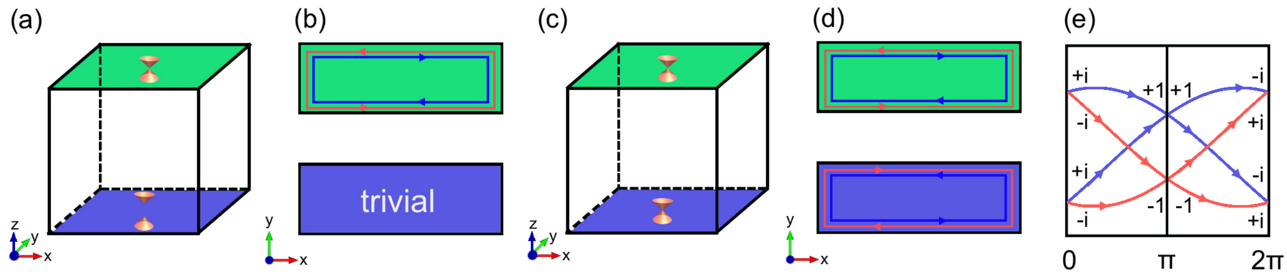

Figure S 16. (a) Schematic demonstration of surface states for BOFTIs in momentum space. Even in the (001) surface, top surface with green color has the metallic surface states, while bottom surface with purple color only has the gaped surface states or bulk states. (b) Spin polarization distributed on the top and bottom surfaces, where the blue (red) branch corresponds to spin-down (up) electrons. (c) Schematic demonstration of surface states for TIs in momentum space. The opposite surfaces in the same direction always have the metallic surface states. (d) Spin polarization distributed on the top and bottom surfaces for TIs. (e) Glide plane $\{g|t\}$ induced Möbius states on a g-invariant-lines. Taken the eigenvalues of g as $g_{\pm} = \pm ie^{-ik/2}$, the eigenvalues of 0, π, and 2π momentum points can be written as $\pm i$, ± 1, and $\mp i$. Then, if one goes around k for 2π, one cannot back to the origin, resulting in the Möbius states.

IX. Surface fermion under perturbations.

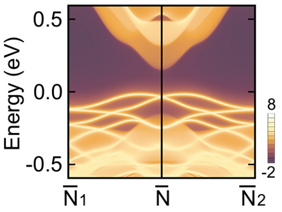

Figure S 17. (001)-surface band structure of Tl_4Se_3Sn calculated using surface Green's functions under perturbation of strain. The Fermi level is set to zero. Although the perturbation does not induce another band inversion, the whole surface states are still lower down. However, the hourglass fermion cannot be broken as long as the glide mirror symmetries are preserved.

X. Detailed crystallographic information and previous growth reports of the eight candidates

TABLE III: Growth method and crystallographic information

Candidates	Growth method	Space group	a (Å)	Wyckoff positions
$TlGaTe_2$	modified Bridgman method [7]	I4/mcm (No.140)	7.02	Tl (4a), Ga (4b), Te (8h)
$InGaTe_2$	Bridgman-Stockbarger method [8]	I4/mcm (No.140)	6.94	In (4a), Ga (4b), Te (8h)
$NaGaTe_2$	melting experiments [9]	I4/mcm (No.140)	6.79	Na (4a), Ga (4b), Te (8h)
$BaPbO_3$	hydrothermal experiments [10]	I4/mcm (No.140)	6.19	O (8h, 4a), Ba (4b), Pb (4c)
InTe	Bridgman–Stockbarger technique [11]	I4/mcm (No.140)	7.09	In (4a, 4b), Te (8h)
Tl_5Te_2Br	melting experiments [12]	I4/mcm (No.140)	9.04	Tl (4c, 16l), Te (8h), Br (4a)
Tl_4Se_3Pb	reaction of the elemental components [13]	I4/mcm (No.140)	8.86	Tl (16l), Se (4a, 8h), Pb (4c)
Tl_4Se_3Sn	Bridgman method [14]	I4/mcm (No.140)	8.81	Tl (16l), Se (4a, 8h), Sn (4c)

* Electronic address: daiy60@sdu.edu.cn
† Electronic address: c.niu@sdu.edu.cn

[1] J. P. Perdew, K. Burke, and M. Ernzerhof, Phys. Rev. Lett. 1996, **77**, 3865 .
[2] G. Kresse and J. Furthmüller, Phys. Rev. B 1996, **54**, 11169 .
[3] G. Pizzi, V. Vitale, R. Arita, S. Blügel, F. Freimuth, G. Géranton, M. Gibertini, D. Gresch, C. Johnson, T. Koretsune, et al., J. Phys. Condens. Matter 2020, **32**, 165902.
[4] A. A. Mostofi, J. R. Yates, Y.-S. Lee, I. Souza, D. Vanderbilt, and N. Marzari, Comput. Phys. Commun. 2008, **178**, 685 .
[5] F. Freimuth, Y. Mokrousov, D. Wortmann, S. Heinze, and S. Blügel, Phys. Rev. B 2008, **78**, 035120.
[6] Q. Wu, S. Zhang, H.-F. Song, M. Troyer, and A. A. Soluyanov, Comput. Phys. Commun. 2018, **224**, 405.
[7] A. Nagat, G. Gamal, and S. Hussein, Cryst. Res. Technol. 1991, **26**, 19.
[8] E. Gojaev, K. Gyul'mamedov, A. Ibragimova, and A. Movsumov, Inorg. Mater. 2010, **46**, 353.
[9] J. Weis, H. Schäfer, and G. Schoen, Z. Naturforsch., B 1976, **31**, 1336.
[10] R. Shannon and P. Bierstedt, J. Am. Ceram. Soc. 1970, **53**, 635.
[11] S. Misra, P. Levinskỳ, A. Dauscher, G. Medjahdi, J. Hejtmánek, B. Malaman, G. J. Snyder, B. Lenoir, and C. Candolfi, J. Mater. Chem. C 2021, **9**, 5250.
[12] D. Babanly and M. Babanly, Russ. J. Inorg. Chem. 2010, **55**, 1620.
[13] T. Malakhovska, M. Y. Sabov, E. Y. Peresh, V. Pavlyuk, and B. Marciniak, Chem. Met. Alloys 2009, pp. 15–17.
[14] I. Barchij, M. Sabov, A. El-Naggar, N. AlZayed, A. Albassam, A. Fedorchuk, and I. Kityk, J. Mater. Sci. Mater. Electron. 2016, **27**, 3901.
